# Individual and community-level predictors of maternal alcohol consumption during pregnancy in Gondar town, Northwest Ethiopia: a multilevel logistic regression analysis

**DOI:** 10.1186/s12884-021-03885-4

**Published:** 2021-06-05

**Authors:** Alemu Earsido Addila, Telake Azale, Yigzaw Kebede Gete, Mezgebu Yitayal

**Affiliations:** 1 Department of Public Health, College of Medicine and Health Sciences, Wachemo University, Hossana, Ethiopia; 2 Department of Epidemiology and Biostatistics, Institute of Public Health, College of Medicine and Health Sciences, University of Gondar, Gondar, Ethiopia; 3 Department of Health Education and Behavioral Sciences, Institute of Public Health, College of Medicine and Health Sciences, University of Gondar, Gondar, Ethiopia; 4 Department of Health Systems and Policy, Institute of Public Health, College of Medicine and Health Sciences, University of Gondar, Gondar, Ethiopia

**Keywords:** Pregnant women, Alcohol consumption, Ethiopia, Multilevel model

## Abstract

**Background:**

Prenatal alcohol consumption is a serious public health concern that is considered as one of the preventable risk factors for neonatal and childhood morbidity and several adverse pregnancy outcomes. This study aimed to determine the individual- and community-level predictors of maternal alcohol consumption during pregnancy in Gondar town, Northwest Ethiopia.

**Methods:**

A community-based cross-sectional study was conducted among pregnant women in Gondar town from 13 June to 24 August 2019. A cluster random sampling technique was used to select 1237 pregnant women. Data collection was carried out using the AUDIT-C pretested standard questionnaire. Bivariable and multivariable multilevel logistic regression analyses were computed to identify predictors of alcohol consumption using the odds ratio, 95% CI, and *p*-value < 0.05.

**Results:**

The prevalence of alcohol consumption during pregnancy was found to be 30.26% (95% CI: 27.74%, 32.91%). The study revealed that pregnant women who have a low knowledge level on harmful effect of alcohol consumption (AOR = 3.2; 95% CI: 1.9, 5.4), positive attitude towards alcohol consumption (AOR = 7.5; 95% 5, 11), history of pre-pregnancy alcohol consumption (AOR = 4.8; 95% CI: 3.4, 6.9), whose partner consume alcohol (AOR = 3.9; 95% CI: 2.5, 6), a perception that alcohol consumption is culturally or socially acceptable (AOR = 3.6; 95% CI: 2.4, 5.3), who were encouraged by their partners to consume alcohol (AOR = 4; 95% CI: 1.9, 8) were significantly associated with pregnancy alcohol consumption. Concerning the community-level characteristics, who had not ever heard/media exposure about the risk of alcohol drinking during pregnancy (AOR = 3; 95% CI: 1.7, 5.5), and who were from low community women’s education attainment (AOR = 4; 95% CI: 2.2, 7.7) were statistically significant predictors of alcohol consumption during pregnancy.

**Conclusions:**

The study revealed that alcohol consumption during pregnancy is prevalent in Gondar town. Both individual- and community-level predictors were found to be associated with alcohol consumption during pregnancy. Policymakers may take into account these predictors for individual and community-based interventions to which our results appear to point.

**Supplementary Information:**

The online version contains supplementary material available at 10.1186/s12884-021-03885-4.

## Background

Different types of alcoholic beverages are produced and consumed worldwide with meals, used to celebrate special occasions, as a social facilitator, and served for medicinal purposes [1]. The health burden of alcohol-related consequences on females is a major public health concern because alcohol drinking among women has steadily been increasing in line with economic development and changing gender roles [2, 3].

Prenatal alcohol consumption is a serious public health concern that is considered as one of the preventable risk factors for neonatal and childhood morbidity and several adverse pregnancy outcomes [4, 5]. Alcohol consumption during pregnancy may cause miscarriage, stillbirth, premature birth, congenital malformations, intrauterine growth retardation, and low birth weight [6–8]. It is also attributed to fetal alcohol spectrum disorders (FASDs), a group of conditions related to alcohol exposure before birth characterized by a range of lifelong irreversible negative health impacts such as physical, behavioral, and intellectual disabilities [9, 10]. Because of this, there is no universally accepted safe amount and time of alcohol consumption during pregnancy, almost all guidelines advocate for pregnant women and women trying to conceive to abstain from any amount of alcohol consumption because it is a known teratogen and causes potential adverse effects on pregnancy outcomes [11–16]. Some studies on the relationship between the risks and the amount of alcohol consumed suggest that low to moderate drinking does not result in the same outcomes as heavy drinking [17, 18].

Based on a systematic review of the WHO Africa Region in 2016, the prevalence of alcohol consumption during pregnancy among the general population was estimated at 2.2% in Equatorial Guinea and 20.5% in Uganda the lowest and highest, respectively while 7.9% in Ethiopia [19]. According to different studies conducted in various regions of Ethiopia, the range of alcohol consumption among pregnant women varied from 4.3% to 34.0% [20–23]. Besides, some reports have shown that alcohol consumption is predominant in Ethiopia. For instance, Ethiopia has been ranked fifth among the ten top local alcohol drinking countries in the world [24].

Alcohol is often consumed in more harmful pattern and has been underestimated in the developing world, like Africa including Ethiopia [25, 26]. Because of the weak regulatory strategies of alcohol production, promotion, and drinking pattern, it is available in every grocery and bar in Africa [27]. In Ethiopia, *“Areki”, “Tella” and “Tej”* are local alcoholic beverages that are produced and accessible everywhere [28]. In Ethiopia, there is an expansion of industrial production and marketing of alcohol which may be driving forces to use alcohol. Health policies in the nation have paid little attention to the risks associated with alcohol consumption during pregnancy. Empirical evidence shows that several women in the study area in particular and Ethiopia in general, consume traditional indigenous and recorded alcoholic beverages while pregnant.

Accordingly, alcohol consumption during pregnancy has serious public health implications in Ethiopia which indicates appropriate policy response by the government and other organizations to design targeted interventions focusing on pregnant women's alcohol use [19]. However, there is a dearth of evidence in Ethiopia on alcohol consumption during pregnancy to give insight or emphasis for policymakers on early detection strategies for the prevention of alcohol use and future directions.

## Methods

### Study design and population

A community-based cross-sectional study was conducted in ten clusters of Gondar town. All pregnant women in Gondar town were a source population, and all pregnant women in the selected kebeles (a small geo-administrative unit that has its boundary) were the study population.

### Study setting and period

The study was conducted in ten kebeles of Gondar town from 13 June to 24 August 2019. Gondar town is located about 727 km far from Addis Ababa, the capital city of Ethiopia. In 2018, according to the Gondar town finance and economic development branch office report, the total population of Gondar town was approximately 338,646 (165, 937 males and 172, 709 females). Of these females, 7454 were estimated to be pregnant women. The town has six sub-cities and 25 kebeles. It has one comprehensive specialized hospital, one polyclinic, and eight health centers [29]. There is one beer factory in the town. Besides, all other types of alcoholic drinks are accessible in the town.

### Sample size determination and sampling procedure

The sample size was determined by EPI INFO version 7.2.1.0 STAT CALC software for cross-sectional study by using a two-populations proportion formula; assumption of a 95% confidence interval (2-sided), 80% power, 31.3% of outcome in the non-exposed group, 41.4% of outcome in the exposed group, and non-exposed to an exposed ratio of 1:1; and considering “marital status” as a predictor factor to bring a difference in two population based on the research conducted in Bahir Dar town [21], and design effect (DEFF) of 1.5 [30], a 10% non-response rate. This gave a sample size of 1237 pregnant women. The study employed a two-level cluster sampling technique to select the study participants. Since the town has 25 kebeles, ten kebeles (clusters) were selected using a simple random sampling technique and all pregnant women from selected clusters within the study period were included in the study.

### Study variables

#### Response variable

The response variable of the study alcohol consumption during pregnancy was categorized as “Yes” or “No”.

#### Explanatory variables

The independent variables were selected based on their significance in previous studies, and classified as individual and community-level factors.

#### Individual-level factors

Individual-level variables included in this category were age group of women, marital status, religion, women’s education, and husbands’ education, family size, occupation, household wealth status, knowledge about the harmful effect of alcohol use, attitude towards alcohol use, depression, social support, parity, number of children, history of pre-pregnancy alcohol consumption, ever heard the risk of alcohol use, the pattern of current pregnancy, a perception that alcohol consumption is culturally or socially acceptable, partner alcohol consumption, and ANC visit.

#### Community-level factors

The aggregate community-level explanatory variables were created by aggregating individual-level covariates with their respective communities. These were computed using the average values of the proportions of individuals in each category of respective variables. The cut-off point for the categorization of the aggregate variables was done as high or low based on the distribution of the proportion or the median values for each community since all aggregates were not normally distributed. The community-level factors were *Kebeles/clusters* (Kirkos, Megenegna, Shewaber, Shiromeda. Medhanialem, Abya-egzi, Keha-Eyesus, Gebrael, Abajale, and Abasamuel), *women’s education attainment in the community*-the median value of the education attainment was secondary and above levels. Then the aggregated clusters were classified into low if the median value of the cluster was below secondary level and high if the median value of the cluster was secondary and above levels, *community media/information exposure* was categorized as low if the proportion of women exposed to media or heard information on the risk of alcohol drinking during pregnancy in the community was 0–56% whereas high if the proportion was 57–100%, *wealth status in the community*-the median value of the wealth index of the household in the area was three. It was categorized as low if the median value of a given community below three and high if the median value of the cluster was greater than or equal to the top three wealth quintiles and *social support in the community*- the median value of the Oslo 3-items social support scale was 11. Accordingly, high if the median value of social support in the cluster was 12 to 14 and low if the median value was 3 to 11.

### Data collection method and instrument

The questionnaire was prepared first in English and then translated into Amharic (local language) to suit local applicability and then back to English to ensure its consistency. The tool was developed by reviewing previous studies of similar objectives [21, 31–36], after that experts’ consultation was sought to ascertain its validity by considering the local situation of the study participants and clinical relevance. Data were collected using structured and pre-tested interviewer (BSc nurses and midwives) administrated questionnaires by face-to-face interview techniques. Data collectors and supervisors were adequately trained on data collection tools, a procedure during data collection, a way how to obtain consent from participants and not to miss any questions in the questionnaire. The Amharic version questionnaire was pre-tested for clarity. It was more validated through a pilot study in 67 respondents in Bahir Dar town which is 180 km far away from the actual study area. The tool was checked for its reliability and validity before actual data collection. When the Cronbach's alpha coefficient/ reliability score 0.7 and above was accepted for internal consistency of the measurement.

The tool contained Alcohol Use Disorder Identification Test-Consumption (AUDIT-C) [35, 37]- the most popular shortened version of the 10-item AUDIT that comprises three items to assess alcohol consumption cross-culturally and identify hazardous drinkers [38, 39]. It is also a brief screening tool that has been used for measuring: frequency of alcohol drinking (any kind of alcoholic beverage), the quantity of alcohol consumed, and frequency of binge drinking (≥ 4 standard drinks on one occasion) of alcohol during pregnancy [33], the Edinburgh Postnatal Depression Scale (EPDS) which has 10 items scored on a scale of 0–3; the score ranging from 0–30 and we used a cut-off point of 13 and above on the scale to identify women with depressive symptoms [40], the Oslo 3-items social support scale, which is considered one of the best predictors of mental health, covering different fields of social support and perceived way of getting assistance from neighbors [41, 42]. The sum ranges from 3 – 14 and the score shows poor support (3–8), moderate support (9–11), and strong support (12–14). Knowledge about alcohol consumption during pregnancy was assessed using 14 questions of 3-point Likert scales (yes, I do not know and no) [31, 34, 36]. The questions were attracted to a value of 1 for correct response or 0 for wrong or do not know the response. Participants’ overall knowledge was categorized using original Bloom’s cut-off point [43–45], as good if the score was between 80 and 100% (12–14 points), moderate if the score was between 60 and 79% (9–11 points), and poor if the score was less than 60% (< 9 points) of a correct answer. Similarly, attitude towards alcohol consumption during pregnancy was evaluated using 11 questions. The 5-point Likert scale from strongly agree to strongly disagree questions were scored an agreement scale of 1 (strongly agree and agree) or 0 (neutral, disagree, and strongly disagree) [31, 34, 35, 46]. The overall level of attitude toward alcohol consumption during pregnancy was categorized using original Bloom’s cut-off point, as positive if the score was 80–100% (9–11 points), neutral if the score was 60–79% (7–8 points) and negative if the score was less than 60% (< 7 points). The socio-economic status of the households (wealth index) was assessed using 16 variables extracted from Ethiopia Demographic and Health Survey 2016 and then Principal Component Analysis was computed to determine it. The internal consistency of the measurement of knowledge and attitude was assured by computing Cronbach alpha coefficient of the pilot study and it was 0.68, and 0.89 for knowledge and attitude, respectively. However, in this study, the Cronbach alpha coefficient was 0.91, and 0.82 for knowledge and attitude, respectively. The questionnaire for knowledge and attitude was attached as supplementary files (supplementary file 1 and supplementary file 2).

### Alcohol consumption measures

The questionnaire was adjusted by considering the local context of alcoholic beverages of alcohol content and drinking containers. Though the amount of alcohol content in a standard drink varies from country to country, we used the WHO's standard for this study, since Ethiopia has no national alcohol policy defining standard alcohol drinks [47]. Based on this, for a standard drink, 12 g of absolute alcohol was assumed which was considered as alcohol consumption. A standard drink was determined by converting local drinks to grams of pure alcohol, and then we specified the amount of pure alcohol per local drink and using local units of measure. Different receptacles were used to measure local drinks, such as ‘tassa’, malekia’ and ‘birille’ for drinks Tella (Ethiopian traditional beer fermented from mostly barley but also with wheat, maize, sorghum, and mixed with ‘Gesho’ [Rhamnusprinioides]) [48], Areki (a whisky-like drink distilled from fermented barley or maize and mixed with ‘[Rhamnusprinioides]) and Tej (a honey wine) respectively. The amount of each drink consumed in ml was then calculated. This value was converted to grams of absolute alcohol by applying a conversion factor and taking into account the percentage of absolute alcohol present in each drink. Accordingly, a standard drink equivalent to 1 bottle beer (330 ml) at 5% x (strength) 0.79 (conversion factor) = 13 g of ethanol; 1 glass wine (140 ml) at 12% × 0.79 = 13.3 g of ethanol; 1 shot (‘malekia’) *areki* (40 ml) at 40% × 0.79 = 12.6 g of ethanol, alcoholic content (30–50%); 1 ‘birille’ *Tej* (200 ml) at 8% × 0.79 = 12.64 g of ethanol, alcoholic content *(*7%- 11%);and 1 “tassa” *Tella/Korofe* (330-500 ml) at 4.5% × 0.79 = 11.73 g of ethanol of alcoholic content (4%—6%) [28, 49, 50].

### Statistical analysis

The questionnaire was double entered and edited into EpiData 3.1.version and then exported to STATA version 14 software packages for analysis. We applied multilevel binary logistic modeling to identify predictors of alcohol consumption during pregnancy. The units at a lower level were individuals (pregnant women) who were nested within communities at a higher level (clusters/ Kebeles). Clusters were considered as random-effects to account for the unexplained variability at the community level. The clustering effect was computed using the Intra-Class Correlation Coefficient (ICC) which is the percentage of variability explained by the higher level.

We carried out bivariable multilevel logistic regression analysis to estimate the crude odds ratios at 95% confidence interval and those explanatory variables with crude odds ratios of *p*-value < 0.25 were considered as a candidate for the adjusted multivariable multilevel logistic regression model. Finally, four models of multivariable multilevel logistic regression analyses were built to estimate the adjusted odds ratios (AOR) by controlling confounders and the extent of random variations between clusters.

### Model building

All four models were built using the stepwise forward method of model building methods and they were fitted xtmelogit command in STATA version 14.0.

- Model I (The null model or the intercept-only model) was fitted without any predictor variables to estimate the clustering effect between-cluster variation and to justify the application of multilevel analysis by computing ICC [51]. It was also served as a benchmark with which other models were compared.
- Model II (individual-level variables) was used to examine whether the variation across communities could be explained by the characteristics of the women residing within that community or not.
- Model III was fitted to assess the impact of community characteristics on the outcome variable (alcohol use during pregnancy).
- Model IV (final model) was fitted to examine the influences of both individual and community-level characteristics simultaneously. In this model, individual and community-level characteristics statistically significant in models II or III were included in the analysis.

The measures of association (fixed-effects) between the likelihoods of alcohol consumption during pregnancy and independent covariates (individual and community variables) were expressed as adjusted odds ratio (AOR), 95% confidence interval, and *p*-value < 0.05 was determined to be a cut point for statistical significance. The random effects were the measures of variation in alcohol use across communities expressed as ICC and proportional change in variance.

Proportional Change in Variance (PCV) was computed with respect to the null model to investigate the relative contribution of individual and community-level factors in explaining alcohol consumption [52]. The log-likelihood and Akaike Information Criterion (AIC) tests were used to estimate the goodness-of-fit of the adjusted final model in comparison to the other models; the lowest AIC in the model was considered as the best fit model. The occurrence of multicollinearity among explanatory variables was ensured using the Variance Inflation Factor (VIF) at a cut-off point of 10 and there was no multicollinearity [53]. The interaction effect of the variables was checked by creating the product term and then a new variable became either statistically significant or not at *p*-value < 0.05.

### Ethical considerations

Ethical clearance was obtained from the Institutional Ethical Review Board of the University of Gondar (R. No.-O/V/P/RCS/05/747/2019) and permission was granted from the Gondar town health department. All the study participants were informed about the objective and importance of the study and verbal informed consent was obtained before conducting data collection. After taking the necessary information, participants were counseled about the risks of alcohol drinking during pregnancy. Besides, women who were engaged in heavy or problematic drinking were encouraged to develop health-seeking behavior and proper linkage was established with health facilities.

## Results

### Individual-level characteristics of study participants

#### Socio-demographic and economic characteristics of the respondents

A total of 1216 out of 1237 pregnant women participated in the study with a response rate of 98.3%. The majority of the pregnant women were in the age group between 25 and 34 years (66.3%) with a mean age of 27.18 (95% CI: 26.93, 27.43). Most of the participants were married 1164 (95.7%), orthodox 1023 (83.96%), Amhara by ethnicity 1183 (97.29%), housewives 672 (55.3%), and had secondary and above education 442 (61.76%). The proportion of participants was nearly equal among the wealth quintiles, low 405 (33.31%), middle 406 (33.38%), and high 405 (33.31%) (Table 1).

**Table 1:** Socio-demographic and economic characteristics of study participants in Gondar town, Northwest Ethiopia, 2019 (*n* = 1216)

Variables	Alcohol consumption		Total (%)	(*p*-value)
	Yes (%)	No (%)		
**Age group (years)**				0.74
15–24	95 (31)	211 (69)	306 (25.16%)	
25–34	243 (30)	563 (70)	806 (66.28%)	
≥ 35	30 (28.8)	74 (71.2)	104 (8.55%)	
**Marital status**				0.54
Married	356 (30.6)	808 (69.4)	1164 (95.72%)	
Single/divorced/separated/widowed	12 (23)	40 (77)	52 (4.28%)	
**Religion**				< 0.68
Orthodox	314 (30.7)	709 (69.3)	1023 (84.12%)	
Muslim	50 (28)	128 (72)	178 (14.64%)	
Protestant	4 (26.7)	11 (73.3)	15 (1.23%)	
**Ethnicity**				0.04
Amhara	352 (29.8)	831 (70.2)	1183 (97.29%)	
Others	16 (48.5)	17 (51.5)	33 (2.71%)	
**Family size**				0.54
1–2	142 (31)	314 (69)	456 (37.50%)	
3–4	157 (28)	404 (72)	561 (46.13%)	
≥ 5	69 (34.7)	130 (65.3)	199 (16.37%)	
**The education level of respondents**				0.004
No formal education	102 (42)	140 (58)	242 (19.90%)	
Primary education (1–8)	63 (28.3)	160 (71.7)	223 (18.34%)	
Secondary education (9–12)	133 (30)	309 (70)	442 (36.35%)	
Tertiary education (above 12)	70 (22.7)	239 (77.3)	309 (25.41)	
**The education level of husbands**				0.001
No formal education	99 (38.8)	156 (61.2)	255 (20.97%)	
Primary education (1–8)	63 (33)	126 (67)	189 (15.54%)	
Secondary education (9–12)	117 (31)	259 (69)	376 (30.92%)	
Tertiary education (above 12)	89 (22)	307 (78)	396 (32.57%)	
**Occupation**				0.03
Housewife	230 (34.2)	442 (65.8)	672 (55.26%)	
Employed in any organization	75 (28.4)	190 (71.6)	264 (21.79%)	
Private business	30 (19.5)	124 (80.5)	154 (12.66%)	
Daily-laborer	20 (32.7)	41 (67.3)	61 (5.02%)	
Others	13 (20.3)	51 (79.7)	64 (5.26%)	
**Household wealth index**				0.68
Poorest	80 (32.6)	165 (67.4)	245 (20.15%)	
Poor	76 (30.5)	173 (69.5)	249 (20.48)	
Middle	65 (24.2)	203 (75.8)	268 (22.04)	
Rich	71 (33)	143 (67)	214 (17.60%)	
Richest	76 (31.6)	164 (68.4)	240 (19.74%)	

#### Depression and social support history of pregnant mothers

Concerning depression, 162 (13.32%) of the pregnant women had depression; of these, 60 (37.04%) of them were alcohol-drinking pregnant mothers (*Y2* = 0.4, *p*-value = 0.53). The findings of social support as measured using the Oslo 3-items social support scale (OSS-3) were scored 18.75%, 51.64%, and 29.61% as poor, moderate, and strong, respectively in overall pregnant women (*Y2* = 5.75, *p*-value = 0.017).

#### Obstetric and medical history of pregnant women

Among the participants, 745 (61.27%) of them were in the third trimester. For 527 (43.34%) of the women, the current pregnancy was their first pregnancy. Most of the pregnant women (44.08%) had experienced one or two children and 26 (2.14%) of them had five or more children. Pregnancies were planned in more than four in five (82.48%) women of the sample. Concerning antenatal care (ANC) follow-up, the majority 1,101 (90.54%) of the pregnant mothers followed ANC. Overall, 111 (9.13%), and 18 (1.48%) of the pregnant women had practiced a history of abortion, and hypertension, respectively.).

#### Knowledge and attitude of study participants on alcohol consumption

The study revealed that 534 (43.91%) of the pregnant mothers had not heard any information about the risk of alcohol drinking during pregnancy, and from 1,101 study participants who had ANC follow up, only 168 (15.26%) were informed about the risks of drinking alcohol by health care providers. The mean score of the participants' knowledge on the risks of alcohol consumption during pregnancy was 5.22 ($\pm$ 4.5 SD), and 891 (73.27%) participants had low overall knowledge on the effect or risk of alcohol consumption during pregnancy. The overall mean score for attitude towards alcohol consumption was 8.05 ($\pm$ 2.3 SD), and 660 (54.28%) participants had a negative attitude towards alcohol consumption (Table 2).

**Table 2:** Knowledge and attitude of study participants on alcohol consumption during pregnancy in Gondar town, Northwest Ethiopia, 2019 (*n* = 1216)

Variables	Alcohol consumption		Total (%)	*p*-value
	Yes (%)	No (%)		
**Ever heard the risk of alcohol drinking during pregnancy**				< 0.001
Yes	133 (19.5)	549 (80.5)	683 (56.09%)	
No	235 (44)	299 (56)	533 (43.91%)	
**Source of information among heard (** ***n*** ** = 683)**				0.08
Television	40 (33.4)	70 (66.4)	110 (16.11%)	
Radio	12 (34.2)	23 (63.8)	35 (5.12%)	
Health professional	53 (31)	118 (69)	171 (25.04%)	
Friends/family	32 (24.8)	97 (75.2)	129 (18.89%)	
Two and above sources	61 (25.6)	117 (74.4)	238 (34.85%)	
**Informed the risk of alcohol consumption at ANC visit (** ***n*** ** = 1,101)**				0.34
Yes	36 (21.4)	132 (78.6)	168 (15.26%)	
No	294 (31.5)	639 (68.5)	933 (84.74%)	
**Level of knowledge**				< 0.001
Low	306 (34.3)	585 (65.7)	891 (73.3%)	
Moderate	33 (27)	89 (73)	122 (10.03%)	
High	29 (14.3)	174 (85.7)	203 (16.69%)	
**Attitude towards alcohol consumption**				< 0.001
Negative	105 (15.9)	555 (79.1)	660 (54.28%)	
Neutral	45 (35)	83 (65)	128 (10.53%)	
Positive	218 (51)	210 (49)	428 (35.20%)	
**History of pre-pregnancy alcohol consumption**				< 0.001
Yes	217 (47.7)	238 (52.3)	455 (37.42%)	
No	151 (19.8)	610 (80.2)	761 (62.58%)	
**Partner alcohol consumption**				< 0.001
Yes	329 (40.5)	484 (59.5)	813 (66.86%)	
No	39 (9.7)	364 (90.3)	403 (33.14%)	
**Partner encouragement to alcohol consumption**				< 0.001
Yes	38 (70.4)	16 (29.6)	54 (4.44%)	
No	330 (28.4)	832 (71.6)	1,162 (95.56%)	
**Peers or family encourage to alcohol consumption**				0.003
Yes	30 (51.7)	28 (48.3)	58 (4.77%)	
No	338 (29.2)	820 (70.8)	1,158 (95.23%)	
**The perception that alcohol consumption is culturally or socially acceptable**				< 0.001
Yes	150 (52.6)	135 (47.4)	285 (23.44%)	
No	218 (23.4)	713 (76.6)	931 (76.56%)	

#### Community-level characteristics of study participants

This study indicated that 880 (72.37%) of the study participants were from high wealth status community, and only 439 (36.10%) had high community social support. The likelihood of alcohol consumption during pregnancy was significant among the clusters (Kebeles) (x^2^ = 109.73, *p*-value < 0.001). In comparison to other clusters, 87 (23.64%) pregnant women who were living in Shewaber cluster were more likely to consume alcohol. Also, exposure to mass media/information, women’s education attainment, and social support in the community were statistically significant community-level factors in bivariable multilevel logistic regression analysis (Table 3).

**Table 3:** Community-level characteristics of study participants in Gondar town, Northwest Ethiopia, 2019 (*n* = 1216)

Community-level characteristics	Alcohol consumption		Total (%)	*p*-value
	Yes (%)	No (%)		
**Kebele of residence**				< 0.001
Kirkos	19 (15.4)	104 (84.6)	123 (10.12%)	
Megenegna	82 (48.5)	87 (51.5)	169 (13.90%)	
Shewaber	87 (47.3)	97 (52.7)	184 (15.13%)	
Shiromeda	11 (6.7)	153 (93.3)	164 (13.49%)	
Medhanialem	21 (34.4)	40 (63.6)	61 (5.02%)	
Abya-egzi	42 (29.8)	99 (70.2)	141 (11.60%)	
Keha-Eyesus	19 (21)	72 (79)	91 (7.48%)	
Gebrael	26 (41.9)	36 (58.2)	62 (5.10%)	
Abajale	36 (40)	54 (60)	90 (7.40%)	
Abasamuel	25 (19.1)	106 (80.9)	131 (10.77%)	
**Wealth index in the Community**				0.03
Low	149 (44.3)	187 (55.7)	336 (22.53%)	
High	219 (24.9)	661 (75.1)	880 (77.47%)	
**Community women’s education attainment**				0.002
Low	126 (46)	148 (54)	274 (22.53%)	
High	242 (25.7)	700 (74.3)	942 (77.47%)	
**Community social support**				0.004
Low	298 (38.4)	479 (61.6)	777 (63.90%)	
High	70 (16)	369 (84)	439 (36.10%)	
**Community mass media/information exposure**				0.017
Low	212 (39.4)	326 (60.6)	538 (44.24%)	
High	156 (23)	522 (77)	678 (55.76%)	

#### The prevalence of alcohol consumption during pregnancy in Gondar town

The study showed that 30.26% (95% CI: 27.74%, 32.91%) of study participants reported taking alcohol during the current pregnancy. Besides, 284 (77.17%) study participants used to drink alcoholic beverages in the first trimester. In relation to the amount of alcohol consumption on a single occasion, most 262 (71.20%) of them consumed one or two standard drinks, some 98 (26.63%) had three or four drinks, and a few 8 (2.17%) of the participants had five or more standard drinks. The study indicated that, among alcohol users, most of the study participants consumed *Tella* 197 (69.37%) followed by beer/draft 55 (19.37%), wine/*Tej* 9 (3.17%), and two or more drinks 23 (8.10%) in their first trimesters. On the other hand, 151 (53.55%), 78 (27.66%), 16 (5.67%), and 37 (13.12%) of the respondents consumed *Tella,* beer/draft, Areki/Tej/wine/whisky, and two or more different drinks in their second trimester, respectively. Finally, *Tella* 65 (53.28%), beer/draft 34 (27.87%), Areki/Korofe/wine 6 (4.92%), two or more different drinks 17 (13.93%) were consumed in their third trimester.

#### Multivariable multilevel logistic regression analysis of alcohol consumption during pregnancy

The intra-class correlation (ICC) in the empty model (Model I) for alcohol consumption during pregnancy was 14.3% (95% CI: 6%, 31%). This implied that 14.3% of the total variance in alcohol consumption during pregnancy was attributed to differences across the clusters or community-level factors. At individual-level variables (Model II) ever heard the risk of alcohol drinking during pregnancy, knowledge about the harmful effect of alcohol consumption during pregnancy, attitude towards alcohol use, partner alcohol consumption, partner encouragement to alcohol consumption, history of pre-pregnancy alcohol consumption, and cultural or social acceptance of alcohol use were statistically significant factors with alcohol consumption during pregnancy. The ICC in Model II depicted that 18% of the variation in women’s alcohol use was attributable to differences across communities. At community-level factors (Model III) the study showed that there was variation in the likelihood of having maternal alcohol drinking during pregnancy across communities, and this variation was significant (τ = 0.16, *p* < 0.001). The study revealed that residing in communities of exposure to mass media/information about the risk of alcohol consumption while pregnant and women's education attainment of the community had a statistically significant association with alcohol consumption during pregnancy. Finally, individual-level and community-level factors were simultaneously computed to predict alcohol consumption during pregnancy in the combined model. In this model, the study indicated that there was a statistically significant variation in the odds of having maternal alcohol drinking during pregnancy between communities (τ = 0.10, *p*-value < 0.001). About 82% of alcohol consumption during pregnancy in clusters was explained in the final model.

After controlling for other individual and community-level factors, women who had low knowledge levels on the harmful effect of alcohol consumption during pregnancy were 3.2 times (AOR = 3.2; 95% CI: 1.9, 5.4) more likely to drink alcohol compared to women who had a high level of knowledge. Mothers who had positive and neutral attitudes toward alcohol consumption were 7.5 times (AOR = 7.5; 95% 5, 11) and 3 times (AOR = 3; 95% CI: 1.9, 5.2) more likely to drink alcohol compared to women who had a negative attitude, respectively. The odds of alcohol consumption during pregnancy were 3.9 times (AOR = 3.9; 95% CI: 2.5, 6) higher among women whose partners had drunk alcohol compared to women whose partners had not drunk alcohol. Women who had a history of pre-pregnancy alcohol consumption were 4.8 times (AOR = 4.8; 95% CI: 3.4, 6.9) more likely to consume alcohol compared to mothers who had not a history of alcohol consumption. Similarly, the odds of having alcohol use among pregnant women who were encouraged by their partners were 4 times (AOR = 4; 95% CI: 1.9, 8) higher than women who were not encouraged. Women who had a perception that alcohol consumption is culturally or socially acceptable were 3.6 times (AOR = 3.6; 95% CI: 2.4, 5.3) more likely to drink alcohol compared to their counterparts. Finally, after keeping for the contribution of other variables, women who were residing in low social support community were 1.7 times (AOR = 1.7; 95% CI: 0.9, 3.2) more likely to consume alcohol compared to women who were from high social support community at the margin of statistical significance. Women who have not ever heard or not been exposed to mass media about the risk of alcohol drinking during pregnancy were 3 times (AOR = 3; 95% CI: 1.7, 5.5) more likely to consume alcohol than ever heard. The odds of having maternal alcohol consumption while pregnant who were from a community with the low education attainment of women were 4 times (AOR = 4.14; 95% CI: 2.2, 7.7) higher compared to their counterparts (Table 4).

**Table 4:** Multivariable multilevel logistic regression analysis of predictors of alcohol consumption during pregnancy in Gondar town, Northwest Ethiopia, 2019

Variables	COR (95% CI)	Model I (Null model)	Model II AOR (95% CI)	Model III AOR(95% CI)	Model IV AOR(95%CI)
**Level of knowledge**					
Low	3.4 (2.2, 5.2)		3.1 (1.9, 5.3)		3.2 (1.9, 5.4)
Moderate	2 (1.2, 3.8)		1.4 (0.7, 3.0)		1.5 (0.7, 3.2)
High	1		1		1
**Attitude towards alcohol consumption**					
Negative	1		1		1
Neutral	3.7 (2.3, 5.9)		2.8 (1.7, 4.6)		3 (1.5, 5.2)
Positive	9.5 (6.7,13.4)		6.3 (4.2, 9.5)		7.5 (5, 11)
**History of pre-pregnancy alcohol consumption**					
Yes	5.1 (3.8, 6.9)		4.9 (3.44, 7.2)		4.8 (3.4, 6 .9)
No	1		1		1
**Partner alcohol use**					
Yes	5.9 (4, 8.6)		3.9 (2.44, 6.08)		3.9 (2.5, 6)
No	1		1		1
**Partner encouragement to alcohol use**					
Yes	6.4 (3.4, 12)		3.5 (1.64, 7.4)		4 (1.9, 8)
No			1		1
**The perception that alcohol consumption is culturally or socially acceptable**					
Yes	4.7 (3.4, 6.5)		3.6 (2.4, 5.4)		3.6 (2.4, 5.3)
No	1		1		1
**Community women’s education attainments**					
Low	3.3 (1.5, 7.2)			2.6 (1.6, 5.1)	4 (2.2, 7.7)
High	1			1	1
**Ever heard/media exposure about the risk of alcohol drinking during pregnancy**					
Low	2.5 (1.2, 5.5)			2.2 (1.01, 4.9)	3 (1.7, 5.5)
High	1			1	1
**Community social support**					
Low	2.9 (1.4, 6)			1.5 (0.8, 3.2)	1.7 (0.9, 3.2)
High	1			1	1
**Random-effects**					
Community variance		0.55	0.71	0.16	0.10
ICC (%)		14.3	18	5	3
PCV (%)		Ref	-	71	82
**Model fitness**					
AIC		1391.2	975.84	1381.79	946.16
Log-likelihood		-693.5836	-459.9229	-684.8936	-460.1313

## Discussion

The study revealed that alcohol consumption during pregnancy is prevalent in the study area. The present study reported that the overall prevalence of alcohol consumption during pregnancy was found to be 30.26%. The result of combined multivariable multilevel logistic regression analysis implied that alcohol consumption during pregnancy was statistically associated with knowledge about the harmful effect of alcohol consumption during pregnancy, attitude towards alcohol consumption, partner alcohol consumption, partner encouragement to alcohol consumption, pre-pregnancy alcohol consumption, and a perception that alcohol consumption is culturally or socially acceptable among individual-level factors; and residing in communities with social support, ever heard or exposed to mass media about the risk of alcohol drinking during pregnancy, and women education attainment of the community were significantly associated community-level factors with alcohol consumption during pregnancy.

According to the result of the respective literature, alcohol consumption among pregnant women ranged from 8.1% [22] to 59.28% [54]. The prevalence of this study is higher than studies that have been reported in Southern Ethiopia [22], Burkina Faso [55], Zambia [56], Republic of Congo [57], Uganda [58, 59] South Africa [60–62], and Tanzania [63]. On the other hand, it is comparable with studies conducted in South Africa [64], and DR Congo [65]. However, this finding is lower than the finding from Bahir Dar, Ethiopia [21], and Nigeria [54]. The high heterogeneity of drinking practices among pregnant women reported by previous studies might be possibly related to cultural beliefs, health policy, difference in methodology, social norms, health service-related factors like quality of health services, and/or alcohol use screening tools variation. Despite these differences, the finding of this study showed that many pregnant women in the study area continue to drink alcohol. Finding the accurate prevalence and amount of alcohol consumption during pregnancy is very challenging since under-reporting is common because of social desirability bias, religious beliefs, recall bias, and seasonal and geographic variations [66, 67]. Almost 10% and 5% of the pregnant women consumed alcohol 2 to 4 times a month and 2 to 3 times a week, respectively. ‘*Tella’* was primarily practiced alcoholic beverage followed by beer/draft in pregnant mothers at all trimesters. In this study, the prevalence of alcohol use was relatively more prominent than in other Africa countries. The reason might be the weak regulatory mechanism of alcohol production, promotion, and drinking pattern; also traditional alcoholic beverages are culturally or socially acceptable and easily accessible with low cost [27, 48]. This result directly evidences that many pregnant women contradict to alcohol guidelines which advising complete abstinence from alcohol use during pregnancy [11, 68].

The odds of having alcohol consumption among pregnant women who had a low knowledge level about the harmful effect of alcohol use while pregnant were 3.2 times higher than compared to women who had a high level of knowledge. This means women who knew the harmful effects of alcohol use on fetuses and mothers were less likely to consume alcohol during pregnancy. This finding was consistent with the studies conducted in an urban and rural area of South Africa [64] and in South-Eastern Nigeria [69]. This might be due to the reason that having sufficient awareness of the risk of alcohol consumption during pregnancy could influence not to drink alcohol and contributes to an individual’s decision-making process. It is also a fact that knowledge for specific activities is the key factor to start behaving and keeping it continuously.

Similarly, it had also revealed that women’s attitudes towards alcohol consumption had an association with their alcohol use. Pregnant women who had a positive and neutral attitude towards alcohol consumption were 7.5 and 3 times more likely to consume alcohol compared to mothers who had a negative attitude, respectively. This might be because participants’ attitudes toward alcohol use were markedly dependent on their knowledge of the harmful effect of alcohol consumption. It could be predicted that as their knowledge increases, the women will become more negative regarding alcohol use. On the other hand, the result of the analysis had shown that a statistically significant association between the history of pre-pregnancy alcohol consumption and maternal alcohol intake during pregnancy. The odds of having alcohol use among pregnant mothers who had a history of pre-pregnancy alcohol consumption were around 4.8 times more than counterparts. This finding was in agreement with similar studies carried out in Southern Ethiopia [22], Dodoma Region Tanzania [63], South Africa [61], and Nigeria [69]. The probable explanation might be individuals who were exposed to alcohol use before their current pregnancy could adhere to alcohol use due to the development of alcohol abuse. Furthermore, most of the women who experienced pre-pregnancy alcohol use; over time it may become habits that are not easily changed during pregnancy, and that makes it difficult for them to cease abruptly after becoming pregnant. The result of this study highlights the significant need for preconception counseling and intervention with women of childbearing age in order to avoid the adverse effect of alcohol drinking during pregnancy.

Furthermore, this study identified that having a male partner who drinks alcohol predicted their spouse drinking during pregnancy. Women who had their partners drank alcohol were 3.9 times more likely to report drinking than their counterparts. This evidence was supported by studies conducted in Bahir Dar, Ethiopia [21], Dodoma Region Tanzania [63], South Africa [64], and Kampala, Uganda [58]. Moreover, a result of a systematic and meta-analysis which was conducted in Sub-Saharan Africa revealed that male partners drinking behavior is one of the risk factors for women to intake alcohol while pregnant [70]. The probable explanation might be the fact that many cohabiting partners have common behavior of substance use and they shared similar experiences of lifestyles. Besides, partners are usually an essential role model for spouses to decide to drink, and sometimes they can be invited to drink, and becomes difficult for them to resist the invitation. The findings also revealed that perceiving cultural or social acceptance of alcohol consumption was proved to have a significant association. A likelihood of having alcohol consumption during pregnancy among those who had a perception that alcohol consumption is culturally or socially acceptable was 3.6 times more likely to consume compared to women who didn’t perceive it. The association might be due to the fact that culture plays an important role in molding people and creates a set of communal beliefs and ways of thinking [38]. The culture people are born into will influence people's eating and drinking behavior, such as what people eat and drink, and when people eat and drink individuals. Thus, having a perception of cultural or social acceptability of alcohol consumption encourages women to drink freely in the environment.

We also found that clusters in which the women live had an effect on women’s alcohol consumption, independent of their individual factors. For instance, alcohol consumption had been associated with the education attainment of women in a specified community. The results verified that mothers from low community women’s education had higher odds of alcohol drinking than women from high education attainment communities. This result was concord with the finding that had been reported in prior studies [69, 71, 72], but it was contradicted with other studies [21, 63]. The plausible explanation could be women who live in a highly educated females community have greater access to exposure to maternal health-related information thus enabling them to seek appropriate health care services and have the high decision-making power to take actions regarding their health. Additionally, educated women have a higher possibility to get important health-related information through reading different magazines and better catch health messages delivered through different media sources and share acquired information to their neighborhoods or others. The existence of more educated women in a community with good health-seeking behavior seems to influence the health practices of the other women in their community, either positively or negatively. The possible reason for the discrepancy with other studies might be that the community women's education was considered as a community-level variable in this study, whereas women's education was viewed as an individual-level factor in previous studies. The odds of alcohol consumption among pregnant women who have not ever heard or not media exposure about the risk of alcohol drinking during pregnancy were higher 3 times compared to their counterparts. The possible explanation for this correlation might be associated with having adequate information or exposed to different media channels related to the adverse effects of alcohol use during pregnancy supposed less likely to drink alcohol. Another reason might be the easy access of some respondents to health care facilities as well as the substantial variation of urban health extension workers in the clusters. Finally, women residing in communities with low social support were also found to have a higher likelihood of alcohol consumption than women residing in communities with a high rate of social support at the margin of statistical significance. This finding was in line with the previous study done in Debre Berhan, Ethiopia [23]. The high social support at the community level may shine the familiarity of the community about maternal health services and the health service utilization of women in the cluster which positively plays an important role in influencing other women's health-seeking behavior. Indeed, as women living in the same neighborhood share commonly related influences, they tend to experience similar alcohol consumption behavior.

### Strengths and limitations of the study

To the best of our knowledge, this study is one of the very few studies in the Ethiopian pregnant women to determine the individual and community level predictors of alcohol consumption during pregnancy using a well-standardized tool and including different potential predictor covariates. For ethical reasons, participants who were involved in binge drinking were linked with nearby health facilities in addition to proper counseling. Regarding methodological strength, a two-level mixed-effects logistic regression was used to correct for the biases in parameter estimates resulting from clustering and explain the between-cluster differences simultaneously. Despite its strengths, this study has some limitations. Because of the nature of the study design, we could not ascertain the causal relationship between alcohol consumption during pregnancy and individual-level and community-level factors. The prevalence of alcohol consumption might be underestimated because of self-reporting which could be prone to social desirability and recall bias. Our analyses depended entirely on self-reports; therefore, we are unable to guarantee responses without a foundation to our questionnaire. We used cluster as the secondary level variable in our definition of a community. However, a cluster (kebele) could not have an accurate geographic boundary and may not represent an actual community. We created community-level factors by aggregating individual data into cluster values; this may not directly capture data that describe the clusters.

### Implications

The high prevalence of alcohol consumption during pregnancy could suggest that every woman trying to conceive and pregnant women should get comprehensive interventions and strategies to tackle the burden of the problem through adolescent and youth reproductive health services (AYRH), antenatal care, and other mechanisms. Screening pregnant women for alcohol consumption is an important activity to target specific interventions because such alcohol use during pregnancy will certainly negatively impact the health and functioning of the women and their infants. A great effort should be done to improve the knowledge of women about the adverse effect of alcohol use during pregnancy. Women should be informed that the harmful effects of alcohol use during antenatal visits and support should be given on abstain from drinking as a part of routine women's health care. Special advice has to be focused on partners of pregnant women concerning not to cooperate on women's alcohol use. In recognition of the fact that past drinking is the best predictor of pregnancy alcohol use, health care workers have to advise any female of reproductive age to abstain from alcohol consumption. Furthermore, screening of maternal alcohol consumption should be integrated with maternal health services and appropriate action has to be taken.

## Conclusions

Alcohol consumption while pregnant is a public health concern. The study revealed that alcohol consumption during pregnancy was prevalent in the study area.

Our findings further indicate that a need to expand knowledge about the harmful effect of alcohol consumption during pregnancy, social support, education attainment of women in the community, mass media exposure on the risk of prenatal alcohol. Convincing men not to support the alcohol use of their spouses and involving partners in maternal health services are likely to be the most salient factors to abstain from alcohol consumption during pregnancy. Additionally, one should consider the role of the socio-cultural environment even beyond the choice of women in alcohol use.

There were considerable community variations in the outcome variables even after controlling for the effects of both individual and community characteristics representing the occurrence of unobserved factors. Further researches are required that helps to identify these unexplained factors including other variables.

## Supplementary Information

**Additional file 1.**

**Additional file 2.**

## Data Availability

The datasets used and/or analyzed during the current study are available from the corresponding author on reasonable request.

## Abbreviations

AIC: Akaike Information Criterion
ANC: Antenatal care
AUDIT: Alcohol use disorders identification test
AUDIT-C: Alcohol use disorder identification test-consumption
FAS: Fetal alcohol syndrome
FASD: Fetal alcohol spectrum disorder
ICC: Intra-class correlation
PCV: Proportional change in variance
VIF: Variance inflation factor
