# Supplementary material for: Individual and community-level predictors of maternal alcohol consumption during pregnancy in Gondar town, Northwest Ethiopia: a multilevel logistic regression analysis
Source: BMC Pregnancy Childbirth. 2021 Jun 5;21:419. doi: 10.1186/s12884-021-03885-4 (PMC8180107; doi:10.1186/s12884-021-03885-4)
Supplement: Supplementary file 1 — Additional file 1. [file 12884_2021_3885_MOESM1_ESM.docx]

**Supplementary files:**

**Supplementary file 1**: **Questionnaire to assess knowledge level of pregnant women on the effect of alcohol consumption during pregnancy or the unborn child**

| **No** | **Questions** | **Responses** | **Remarks** | |
| --- | --- | --- | --- | --- |
|  | Does alcohol consumption during pregnancy risk for low birth weight? | 1. Yes 2. Not sure 3. No | |  |
|  | May alcohol consumption during pregnancy cause brain damage? | 1. Yes 2. Not sure 3. No | |  |
|  | Does alcohol consumption during pregnancy baby addicted/experiences withdrawal | 1. Yes 2. Not sure 3. No | |  |
|  | Does alcohol consumption during pregnancy cause birth defects/ deformities? | 1. Yes 2. Not sure 3. No | |  |
|  | Does alcohol consumption during pregnancy risk for mental disorders? | 1. Yes 2. Not sure 3. No | |  |
|  | Does alcohol consumption during pregnancy risk for premature birth? | 1. Yes 2. Not sure 3. No | |  |
|  | May alcohol consumption during pregnancy risk for delayed development of offspring? | 1. Yes 2. Not sure 3. No | |  |
|  | May alcohol consumption during pregnancy risk for growth problems? | 1. Yes 2. Not sure 3. No | |  |
|  | Does alcohol consumption during pregnancy risk for learning disabilities? | 1. Yes 2. Not sure 3. No | |  |
|  | Does alcohol consumption during pregnancy risk for lower IQ? | 1. Yes 2. Not sure 3. No | |  |
|  | Does alcohol consumption during pregnancy risk for cranial/facial deformities? | 1. Yes 2. Not sure 3. No | |  |
|  | Does alcohol consumption during pregnancy risk for behavioral Problems? | 1. Yes 2. Not sure 3. No | |  |
|  | Does alcohol consumption during pregnancy risk for miscarriage | 1. Yes 2. Not sure 3. No | |  |
|  | Does alcohol consumption during pregnancy risk for stillbirth? | 1. Yes 2. Not sure 3. No | |  |
