# Supplementary material for: Individual and community-level predictors of maternal alcohol consumption during pregnancy in Gondar town, Northwest Ethiopia: a multilevel logistic regression analysis
Source: BMC Pregnancy Childbirth. 2021 Jun 5;21:419. doi: 10.1186/s12884-021-03885-4 (PMC8180107; doi:10.1186/s12884-021-03885-4)
Supplement: Supplementary file 2 — Additional file 2. [file 12884_2021_3885_MOESM2_ESM.docx]

**Supplementary file 2**: **Questionnaire to assess attitude of pregnant women towards alcohol consumption during pregnancy**

|  | I believe that pregnant women should not drink any alcohol. | 1. Strongly agree 2. Agree 3. Neutral/ not sure 4. Disagree 5. Strongly disagree |
| --- | --- | --- |
|  | If I drink alcohol in moderation and I am sensible, then I don't think it affects the fetus. | 1. Strongly agree 2. Agree 3. Neutral/ not sure 4. Disagree 5. Strongly disagree |
|  | If it has a relaxing effect on me, then I don't see there's any harm. | 1. Strongly agree 2. Agree 3. Neutral/ not sure 4. Disagree 5. Strongly disagree |
|  | I think drinking alcohol helps when I am really stressed out, emotionally up and down as I are during pregnancy, occasionally have a glass of wine is good. | 1. Strongly agree 2. Agree 3. Neutral/ not sure 4. Disagree 5. Strongly disagree |
|  | From my previous experience and others in my neighbor, drinking doesn’t cause any problem. | 1. Strongly agree 2. Agree 3. Neutral/ not sure 4. Disagree 5. Strongly disagree |
|  | I consider drinking small amount alcohol during pregnancy has a beneficial effect for development of the fetus’s brain. | 1. Strongly agree 2. Agree 3. Neutral/ not sure 4. Disagree 5. Strongly disagree |
|  | I think if drinking alcohol caused serious problem, health professionals would give great attention; therefore I consider it doesn’t cause much problem. | 1. Strongly agree 2. Agree 3. Neutral/ not sure 4. Disagree 5. Strongly disagree |
|  | For me consumption of alcohol during pregnancy doesn’t cause spontaneous abortion. | 1. Strongly agree 2. Agree 3. Neutral/ not sure 4. Disagree 5. Strongly disagree |
|  | I don’t think that drinking cause serious effect on newborn if you are not addicted. | 1. Strongly agree 2. Agree 3. Neutral/ not sure 4. Disagree 5. Strongly disagree |
|  | I don’t consider that drinking traditional alcohols like *Tella, Arek*i, and others cause problem on birth outcome. | 1. Strongly agree 2. Agree 3. Neutral/ not sure 4. Disagree 5. Strongly disagree |
|  | I imagine that drinking after first trimester doesn’t cause any troublesome on fetus. | 1. Strongly agree 2. Agree 3. Neutral/ not sure 4. Disagree 5. Strongly disagree |
